# Social psychology and life design meet product design: how to craft a purposeful path, creating roadmaps for meaningful career futures

**DOI:** 10.3389/fpsyg.2026.1924139

**Published:** 2026-10-01

**Authors:** Elisabetta Camussi, Francesco Zurlo, Venanzio Arquilla, Maria Luisa Sbarra, Daria Meneghetti, Maria Cristina Ginevra, Sara Santilli, Chiara Annovazzi

**Affiliations:** 1 Department of Psychology, University of Milano-Bicocca, Milano, Italy; 2 Department of Philosophy, Sociology, Education and Applied Psychology, University of Padua, Padua, Italy; 3 School of Design, Polytechnic of Milan, Milano, Italy

**Keywords:** future orientation, life design, product design, smart skills, university course, young adults

## Abstract

Young people are growing up in a complex and constantly changing world. Its continuous transformations pose significant challenges for the entire population but are particularly impactful for young adults. During the transition from tertiary education to the labor market, they often struggle to envision their future and to imagine their professional careers, so reducing their personal well-being. However, actively reflecting on the future enables individuals to explore multiple possibilities, define their goals, and anticipate as well as better manage upcoming challenges. For these reasons, a group of Social Psychologists of the University of Milano Bicocca and Designers of the Polytechnic University of Milano developed a university course entitled Life Design that was launched in the 2018/2019 academic year within the Integrated Product Design program at the School of Design of the Polytechnic of Milano. The present paper describes the Life Design course, outlining for each topic the corresponding theoretical framework, the specifically designed activities, and the expected outcomes in terms of awareness and skills development. The course is grounded in the most recent and well-established theoretical models in vocational guidance psychology, with particular reference to the literature of the Life Design Research Group. Its main purpose is to prepare future graduates to navigate contemporary complexity and ontological uncertainty. The course aims to equip students with both psycho-social theoretical frameworks and related practical tools based on the development of smart skills, enabling them to better understand the socio-economic context, analyze its dynamic characteristics, and effectively respond to its consequences.

## 1 Introduction

According to Zygmunt Bauman (2000), the contemporary world, or post-modern society, is marked by continuous transitions and rapid changes. His well-known “Liquid society” metaphor underlines how transient are present social transformations and developments, in contrast with the slow mutability of past structures: “Change is the only permanence, and uncertainty the only certainty” (Bauman, 2012). Social forms and institutions hardly persist and solidify within liquid societies, which are not able to provide fixed and precise frames to organize life experiences and expectations (Rattansi, 2017). As a response to permanent uncertainty, temporary and time-limited jobs replaced permanent jobs, and the job industry was marked by an extremely flexible organization (Kalleberg et al., 2000). This reflects on individual development in the form of frequent transitions and changes, leading to non-linear careers and professional pathways (Camussi and Annovazzi, 2013; Savickas et al., 2009). In this context, events like the Covid-19 pandemic or the recent or re-upscaled wars afflicting the West, as that between Russia and Ukraine or the conflict between Israel and Palestine, have a global-scale impact and inevitably lead to increased complexity and non-linearity. Covid-19, for example, was an unpredictable event with pervasive and global consequences and a major impact on people’s well-being. The pandemic accelerated changes and transitions both in individual and social lifestyles (Antonicelli et al., 2020). People’s perceptions of uncertainty, insecurity, and vulnerability increased (Dryhurst et al., 2020), resulting in increased pessimism about the future (ISTAT, 2025). People’s capacity to picture and plan future personal and professional paths was negatively influenced because of fears and insecurities about the future that this situation had generated (Camussi et al., 2023a, 2023b). For these reasons, young people and workers now have to face multiple and harder challenges compared to those experienced by past generations. To address and move through complexity, career counseling psychology brings into play a global and holistic approach that may be able to give individuals the tools to develop their ability to adapt to changes (Blustein et al., 2019). Focusing attention on individual adaptability, promoting flexibility, and life-long learning represent a possible solution (Soresi and Nota, 2020). Consequently, smart transversal skills and psychological resources appear to be necessary in this knowledge-based risk society (Santisi et al., 2021). Possessing the hard skills related to “what to do” in a given job or task is increasingly considered a requisite that cannot be taken for granted. On the other hand, smart skills such as problem-solving, collaboration, communication, creativity, flexibility, and adaptability are mostly linked to “how to act,” i.e. ontologically processual and useful despite any change. Also, smart skills will be crucial in the present and future job market because of the acceleration of skill shifts caused by automation and the advent of artificial intelligence. Given this environment, granting young adults, the dynamic and processual knowledge to comprehend present and future contexts, promoting their skills to face complexity and to adapt to change, and generally preserving and boosting their will to design their own lives and careers appears, of primary importance (Maree, 2021). In this paper, we present a novel University course that has been designed and perfected over the years to respond to these challenges.

## 2 Life design framework

Before going deeper into the course structure, we will present the theoretical framework. Life Design is described by the research group that developed the concept (Savickas et al., 2009) as a theoretical framework and intervention approach based on the epistemology of social constructionism. According to this paradigm, the meaning of individuals’ identity comes from social interactions and is co-constructed through discourse. Thus, it allows the construction and the awareness of co-existing multiple identities and possible selves and encourages their exploration to envisage different paths for the future. Indeed, Life Design seeks to stimulate individuals to create, build, and design “life trajectories,” including work careers. This is possible by providing instruments and fostering skills to deal with the complexity and uncertainty of contextual dynamics and causalities. This approach considers the individual with all their life roles in a holistic way, considering the main aspects related to a career choice. Furthermore, from its social constructionist perspective, the importance of the environment is emphasized and, consequently, the contextual aspect is accentuated. Lastly, the framework must be preventive instead of following social changes or intervening at transition times (Savickas, 2005; Savickas et al., 2009). The main goal of Life Design interventions is to foster people’s adaptability, narratability, and activity as means to support them in building realistic and reasonable career paths (Savickas et al., 2009). Career adaptability, the “macro” skill promoted in a Life Design approach, relies on flexibility in responding to developmental tasks and occupational transitions, and it can be seen as a set of psychological resources. It represents the capability of an individual to face changes successfully (Savickas, 2005), and it refers to the coping strategies and the adaptive behaviors used to face those transitions within the social context (Karaevli and Hall, 2006). A Life Design intervention should help individuals develop a set of transversal skills, such as resilience, realistic optimism, and courage, to anticipate and deal with changes and understand context complexity, supporting expectations and aspirations in line with reality (Savickas et al., 2009). Specifically, career adaptability should be the arrival point. This relates to four dimensions: concern, control, curiosity, and confidence. Being concerned requires future orientation and awareness of vocational tasks and occupational transitions to exploit in a positive way. Career control refers to the disposition to manage vocational tasks, understanding the responsibility, and the possibility to construct one’s own career. Curiosity regards the disposition to explore possibilities, produce knowledge, and scan the environment to learn about self and context, to find a fit between self and the work world. Career confidence concerns self-efficacy and is related to problem-solving, stimulating decision-making, and facing complexities. Life Design should encourage the production in an individual’s own words of coherent stories and narrations about their life, projects, career expectations, memories, and future expectancies. The aim is to allow people to formulate and, consequently, create their own system of subjective identities that is congruent with the context in which they interact and imagine their multiple selves in relation to different situations. In this respect, narratability enables the exploration of multiple subjective identities, or life roles, in different environments and helps with a profound reflection on the significance of each situation. This aspect is useful for creating, constructing, and designing personal and professional trajectories. This practice stimulates self-reflection and increases self-awareness from a perspective that is realized when it leads people to move into action, trying out their strategies and gaining more elements to perfect these strategies.

## 3 The life design course

Building on these premises, the Social Psychologists of the University of Milano Bicocca and the Designers of the Polytechnic University have developed a university course within the Integrated Product Design Master’s Degree at the School of Design of the Polytechnic University of Milan. The present article is intended as a descriptive account of the design and implementation of an educational intervention rather than as an empirical evaluation of its effectiveness. Accordingly, references to potential educational benefits are grounded on the Life Design theoretical framework, in which social psychologists are experts, and in students’ reflective outputs rather than in experimental evidence. Conceived and structured within the scientific framework of social and counseling psychology and specifically based on the psychology of career guidance, the initiative translates psychological theories of self-construction, adaptability, and contextual agency into an educational format tailored for design students (Soresi and Nota, 2020; Savickas et al., 2009). The rationale behind this initiative lies in the profound connection between the design profession and current transformations in the labor market. As a field intrinsically oriented towards innovation and change, design requires the integration of psychosocial skills—such as flexibility, adaptability, resilience, problem-solving, and creativity—alongside technical expertise. Moreover, design practice demands the capacity to anticipate and shape future scenarios, an ability closely related to psychological constructs of future orientation and prospection that are central to both Life Design theory and social psychology. The Life Design course was therefore conceived as a psychosocially informed educational intervention aimed at supporting students in navigating uncertainty and developing competencies for lifelong learning and career adaptability. Structured in four-hour sessions, each lesson combines theoretical contributions with experiential and reflective activities, including individual and group assignments. The theoretical components draw on social psychological models of identity construction, agency, and interaction, providing tools to analyze and manage the relational and contextual dimensions of work. Practical activities are designed to foster opportunities for students to develop interpersonal and self-regulatory capacities—such as collaborative problem-solving, conflict negotiation, responsibility-taking, and initiative—and to encourage reflection on hopefulness, optimism, and self-efficacy as protective and developmental psychological resources. Through these experiences, students are encouraged to reflect on and cultivate a more positive and proactive relationship with the future, an approach theoretically consistent with interventions intended to address future-related uncertainty. The course structure reflects these principles through three interconnected modules: (1) social psychology foundations offering a conceptual basis for understanding meaning-making and motivation within social contexts; (2) the Life Design theoretical framework addressing its relevance to contemporary career challenges; and (3) the development of smart skills as transversal psycho-social competencies. Overall, the course exemplifies one possible translation of social and career guidance psychology into design education, with the aim of supporting adaptability, reflexivity, and wellbeing in students preparing for an increasingly complex professional world.

## 4 The outcome/intervention: the creation of a life design personal portfolio

As anticipated, theoretical explanations are constantly accompanied by practice, consisting of educational and experiential moments (Soresi and Nota, 2020). Through practical activation, students can experiment, learn new abilities, and understand their interests (Camussi et al., 2023a, 2023b). Starting from theoretical concepts, related assignments and tasks increase knowledge and skills and encourage reflectivity, discussion, creativity, and curiosity. Furthermore, practice is intended to provide opportunities to strengthen self-efficacy through interaction with others and to support career construction processes (Soresi and Nota, 2020). For these reasons, every lesson involves one or two practical activities based on the related theoretical aspect and conducted by expert psychologists in career guidance. Each activity envisages a theoretical lesson regarding the topics to be practiced. The results and outputs of each student’s activity are gathered into a personal Life Design Portfolio that is revised during *ad hoc* in-presence lessons. The Portfolio itself is in effect the starting point of each exam. Given the nature of this course, these exams are oral. To be admitted, each student is required to submit their Life Design Portfolio to their teachers. At the beginning of the exam, students are encouraged to freely present their Portfolio, with this presentation constituting a first step followed by and guiding the subsequent questions from the examination board. Importantly, the Life Design Portfolio represents something each student primarily develops for themselves: a personal reflective tool that can, if approached seriously, also serve as an alternative form of self-presentation—a sort of “different CV.” It is conceived as a living document to be maintained and progressively enriched over time, reflecting the evolution of the individual’s competencies, aspirations, and self-awareness. Moreover, students are granted freedom in choosing the Portfolio’s graphical form and structure—a feature that anticipates the discussion presented later—which makes it particularly suitable for design students, though equally valuable and adaptable for students from other disciplines. Individual activities allow students to practice and deepen their comprehension of the main theoretical arguments, to start to become involved in creating their future career paths, and to provide opportunities for the development of smart skills. Each activity requires writing down reflections and impressions about the processes involved. Students are thus invited to organize their thoughts and take more time to analyze them, with the Portfolio becoming a tool to better understand and explore both conscious and unconscious dimensions. The following paragraphs will describe each course section with a more in-depth view of both the theoretical and practical themes assessed.

### 4.1 Matching design and psychology and attentive processes

The Life Design course presents ways to match design and social psychology and the reasons why psychological viewpoints can be useful for a designer by first exploring students’ preliminary ideas about this topic. Psychology can provide tools to observe reality and increase awareness about an individual’s strategies to face daily tasks. Learning to reflect on human behavior is necessary to achieve a good design process. During the course, a comparison between Norman and Lewin is provided to exemplify the match between design and psychology. Norman (2013) defined “affordance” as an interaction between an object’s properties and its capacity to be used. In social psychology, human behavior is defined as an interaction between a person and his or her environment (Lewin, 1951). The psychological process involved and underlined in both authors’ theories is attention (Von Wright et al., 1975). After a presentation of the theory regarding voluntary and involuntary attention, the practical task related to this part of the course is to try to design packaging to capture or distract a potential buyer’s attention. This activity aims to put into practice the theoretical concept, deepening awareness about psychological processes and understanding the extent to which psychological theories could help design something impactful and useful.

### 4.2 Stereotypes and prejudice and the design job world

The following section goes into the cognitive processes involved in comprehending and elaborating reality, aiming to increase consciousness about those themes and promote critical awareness. More specifically, the second lesson and its related activity address schemas, stereotypes (Kanahara, 2006), prejudices, and discrimination, explaining their main functions and consequences (Fiske, 1998). Developing awareness of schema is fundamental because of their pervasive influence. The main function of schemas and stereotypes is reality simplification to allow people to rapidly respond to any situation. One main takeaway is comprehending the automatic and unavoidable nature of these psychological processes (Rudman and Glick, 2001), building awareness about them. Acknowledging automatic cognitive processes fosters a mindset geared to identify reality distortion while designing, whether the object is a product or a career choice. For these reasons, the activity related to this part of the course requires students to reflect on the world of design work. The assignment is to find some pictures and words that are meaningfully representative of individuals’ ideas about designers, clients, companies, and design studios. This exercise is intended to encourage students to reflect on their own stereotypes linked to design words and how those stereotypes could influence their points of view, attitudes, and behaviors. The final aim is to contribute to the creation of a more complex idea of social issues. This activity also allows several considerations about how design can perpetuate stereotypes and prejudices rather than create new representations and fight discrimination.

### 4.3 Group theory and group dynamics awareness

Group theory has also exploited starting from classical psycho-social notions. The theoretical lesson first defines the group concept in social psychology and then explains its main features and typologies relating to Role Theory. According to Career Construction Theory, social roles are involved in identifying and constructing different selves (Savickas, 2019). Present complexity inevitably leads to the coexistence of multiple identities and subjective realities, rendering it fundamental to build awareness about the different social roles each person assumes in different moments of their lives. The different types of leadership that can characterize a group are addressed and described. On this basis, activity number 3, called “Me, My Groups and I,” requires students to think about their group membership, choose some of them and arrange the chosen ones into a graph indicating on the two axes the emotional bond to each group and its size. Subsequently, students choose one group and describe it. The analysis requires pointing out the group characteristics—such as whether it was primary or secondary, voluntary or involuntary—as well as the roles played by each member and the leadership styles. This activity is designed to encourage recognition of group dynamics and reflection on role fluidity and changes related to the context, as well as identifying different leadership styles. Additionally, this type of exercise may support greater self-awareness of how people act and behave in a group and the role they could assume according to a specific circumstance. This experience can be applied to both professional and personal contexts.

### 4.4 Global context, sustainability, and data reading and gathering

Considering the importance of using a holistic approach and the complexity of contexts, the next section of the course required various reflections on the global context and an awareness of the biases with which we perceive this context. These arguments prompt students to think about the existence of severe complexities at social levels and to use data to analyze them. Moreover, to encourage a more optimistic and, at the same time, realistic view of the world, as well as a deeper awareness of the biases each person carries when thinking about the global context, the “Gap Minder” (Rosling et al., 2018) questionnaire is used. This questionnaire is composed of 12 or 18 multiple-choice questions with one correct answer for each. The mean number of correct answers by the global population is 3. The reason for this result lies in the fact that people normally have a biased and more negative than necessary view of many global achievements and facts, such as income, access to electricity, access to school, access to vaccines, etc. Experimenting in the first person, the process of finding out the correct answers and the incorrectness of a globally pessimistic vision may lead students to be more aware of their biases and to build a more optimistic approach. Furthermore, with a view to career guidance aimed at building an inclusive and sustainable future, the United Nations 2030 Agenda for Sustainable Development and its 17 goals are presented and discussed (Soresi and Nota, 2020). Given its contextual nature, this section of the course is exemplified in different fundamental themes that emerge from year to year as well as in the more theoretical and processual elements. For example, considerations about climate change, the Covid-19 pandemic, the great resignation, etc. should encourage people to better understand future challenges and develop both a social and environmental match between their interests and sustainability. The role of career counselling becomes crucial in building future trajectories to support change in an inclusive and sustainable perspective (Nota et al., 2019). Lastly, the Portfolio activity for this part is called “Where do you want to work?” and requires students to imagine where they could build their future, creating a visual description of the Nation they could be interested in working in, making use of the given data and graphics about different themes they consider a priority, such as environment, health, economy and work, education and others. This activity aims to train participants to visualize themselves in the future, building a context that fits their future paths and desires. Comparing different situations and countries is intended to encourage consideration of different possibilities and career paths based on real data and research even in a university activity. Learning to imagine the future, together with using publicly available data and information, is a primary goal in a Life Design perspective.

### 4.5 Life design approach and narratability

In this section, the Life Design perspective regarding Career Adaptability, its dimensions of curiosity, control, confidence, and concern, the importance of stories, and the concept of narratability are more explained in-depth. The subsequent Portfolio activity was inspired by an accredited Life Design career counselling tool called “My Career Story” (Savickas and Hartung, 2012), which allows people to experience more positive action-oriented language and fosters reflective capacity and hope (Santilli and Hartung, 2022). Specifically, the activity asks students to describe and represent a hero of their past, thinking about a real or fictional they admired when they were children. The second step of this activity then asks students to elect their all-time favorite book, series, film, or videogame. They have to briefly describe the plot, and, as for the first step, they are asked to write down their reflections. They need to explore and find all the nexuses between themselves, their aspirations, values, skills, fears, weaknesses, strengths, and the elected story. Finally, they have to present a personal motto. Reflecting on their role models may help to underline the traits, attributes, and values that a person can or wants to attribute to themselves. Moreover, electing a personal motto helps to highlight individual coping strategies and the individual’s idea of life. The meaning of this exercise lies in the fact that the narrations of these elements are related to the people who describe them, their values, their purposes, and their goals, as well as the features that underline the formation of their professional identity. In this way, it may be possible to facilitate identification of links between an individual’s past, present, and future, reflecting, on the one hand, on what is really important and, on the other, on what no longer matters.

### 4.6 Smart skills

The following theoretical section focuses on smart skills, based on their fundamental role in career guidance. As mentioned before, in the context of enormous change, constant transitions, and difficulties, transversal competencies are crucial to ensure satisfactory personal and individual development, as well as reward career possibilities. For example, high-hope students have greater problem-solving capacities and coping strategies (Chang, 1998). Reflexivity and hope are interrelated (Santilli and Hartung, 2022); they regard self-awareness as the integration of past, present, and future, fostering the capacity to visualize goals and achievements. Optimism is linked to other adaptive skills (Segerstrom, 2010), to success in both academic and professional careers (Solberg Nes et al., 2009), and to more adaptive decision-making strategies (Magnano et al., 2015). Additionally, Career Adaptability is related to life satisfaction in adolescence (Ginevra et al., 2018), the capacity to manage vocational transitions (Germeijs and Verschueren, 2006), and the future orientation and breadth of vocational interests (Ginevra et al., 2018). For these reasons, smart skills are deepened, exemplified, and defined in their main characteristics and in contrast to hard skills. The activity related to this section is called “My character creation” and requires students to think about their smart skills. They must choose their most representative smart skills and give them a score. Additionally, students have to visually represent the relationship between smart skills and themselves. To complete this exercise, they must reflect deeply on themselves, their characteristics, and the features that are important in their personal and professional lives. Furthermore, they must identify their strengths, as well as their areas for improvement. Related to this part, students also have to answer a self-assessment questionnaire, containing different dimensions: Design Thinking Skills, Career Adaptability (Duarte et al., 2017), and Smart Skills (Kutrzeba, 2018). Personalized private feedback is then sent to students during the following weeks. This personalized restitution is the basis for another Life Design course Portfolio activity in which students actively reflect on the relations between their perceptions and beliefs about themselves and their results, with the aim of encouraging greater self-awareness.

### 4.7 Behavioral styles and communication: assertiveness

The following theoretical part regards the main psycho-social behavioral styles and the associated ways of communication. The features of passive and aggressive behavioral styles are presented (Bocar, 2017), as well as the issue of assertiveness (Pipas and Jaradat, 2010). Examples from everyday life are provided to help students get a more pragmatic view of these concepts. Also, the manipulative or passive/aggressive behavioral style is explained. Lastly, assertiveness is considered in detail, presenting the numerous positive outcomes stemming from it, as well as the myths that surround this skill and the main strategies with which it can be fostered. The fact that these concepts represent simplified styles, each of which is part of every person’s personality, is stressed, as well as the importance of building awareness about these aspects in order to achieve more effective communication and the ability to work in groups. Subsequently, in order to put the different communication styles into practice, students are asked to immerse themselves in different situations. The activity requires the creation of dialogues, using aggressive, passive, and assertive communication for each of the situations presented. This exercise allows students to reflect on their personal use of communication styles and how they manage different styles, considering the context and the person involved in the conversation.

### 4.8 Self-reflection: representing our history and future perspective

Self-reflection is essential for both personal and professional development; it allows individuals to become aware of their own thoughts, emotions, and behaviors, helping to identify strengths and areas for improvement (Ardelt and Grunwald, 2018). It promotes emotional regulation, reduces cognitive biases, and enhances emotional intelligence, leading to more conscious and intentional decision-making (Gerace et al., 2017). In educational settings, reflecting on what and how we learn has been positively correlated with better academic outcomes (Sobral, 2001). In psychotherapy, self-reflection enables practitioners to recognize transference and countertransference and to work ethically and in alignment with their values (Löffler-Stastka et al., 2023). During difficult or stressful times, it fosters resilience, self-awareness, and psychological recovery (Ardelt and Grunwald, 2018). Furthermore, it supports the formation of clearer goals, greater motivation, and better adaptability to change. However, reflection must be guided and constructive, otherwise, it can lead to rumination or destructive self-criticism (Eva and Regehr, 2008). Structuring self-reflection through journals, supervision, or external feedback has been shown to improve its effectiveness (Driessen et al., 2005). In order to work on self-reflection, the Life Design Portfolio is implemented with two activities: My Personal Story Visualization and My Future Project. The first task asks students to create a visual representation of their story from their childhood to their present comprising the most important steps. As for the other Life Design course Portfolio activities, the choice of graphic style is free. The second task asks students to reflect on their professional project and expectations. In this exercise, students have to concretely imagine themselves in the future, building a realistic project, and exploring different ideas and possibilities. More specifically, the request is to delineate and specify different aspects: the main features of the job they would choose, such as the employment field, the proponent, the type of contract expected; the skills already acquired and those still needed, adjusting the work done during the activity “My character creation”; possible and useful future trajectories in terms of training paths and access routes; and project sustainability. These activities represent a good synthesis of what is experienced during the whole course. In fact, these processes require individuals to have awareness of themselves—the skills they have already acquired and those they need to further develop, their passions and values—a good level of self-efficacy and self-esteem, and a realistic idea of the global context and the job market. They are educational and creative activities designed to encourage students to envision, plan, and articulate their vision of themselves and their personal or professional goals for the future. Through guided writing exercises, participants reflect on their aspirations, values, and long-term objectives, transforming abstract ideas into a concrete, actionable plan. The project is designed to foster self-awareness, motivation, and goal-setting skills, while enhancing participants’ writing and communication abilities. It can be adapted for students, job seekers, or anyone interested in personal development.

### 4.9 Life design workshops: CV building, job interview, and group assessment

This part of the course is aimed at providing students with theoretical knowledge and practical skills to better prepare themselves for entering the job world. The process of candidate selection is explained, including the process of CV skimming and the time HR staff spend on this activity. Related to this, the main parts of the CV are illustrated, and for each, strategies and advice are provided to increase the chances of a CV being chosen. Both human and automatic CV selection processes are also assessed. Examples of companies using artificial intelligence to select their personnel are given, along with strategies to better deal with both cases. The following section deals with the job interview, with a coherent focus on both human and automated interviews. The aims of individual interviews are explained and strategies are provided. Moreover, the concepts of group interviews and assessment centers are considered in depth. The last part of this lesson comprises a practical experience of a group assessment process. This section does not require students to include any of their work in the Portfolio. However, students often spontaneously ask for CV advice or a CV revision after working on it during the days following this lesson and before the next section.

### 4.10 Portfolio revisions and final considerations

The course includes moments of revision of the students’ Portfolios prepared by teaching staff. These revisions are an important aspect of the Life Design course. The activities are designed with the aim of promoting an experiential mode of learning, but these moments of revision are useful to deepen the issues addressed. Furthermore, these moments allow students to present their work to concretize their reflections and render explicit some thoughts that could have remained only implicit. The interaction with staffis intended to support the achievement of the stated goals, opening new perspectives and paths based on a more complex idea of the context and greater self-awareness. Following the Life Design paradigm for career intervention, the course touches on several aspects that are useful in the creation of new expectations, trajectories, and ideas resulting from a more complex and articulated worldview. Furthermore, students have the opportunity to reflect on building a future-oriented personal and professional identity that is consistent with their values and aspirations. These activities illustrate how theoretical concepts may be translated into practice with the aim of supporting future reflection, self-efficacy, and the development of smart skills (Camussi et al., 2023a, 2023b). The resulting Portfolio is intended to serve as a reflective tool for students to reflect on themselves, their competences, their needs, their goals, while learning to consider different contexts, at a global and social level.

## 5 Discussion/conclusion

In conclusion, the introduction of the Life Design course within the Integrated Product Design Master’s Degree at the Polytechnic of Milano constitutes an innovative educational initiative grounded in the scientific framework of Social and Counseling Psychology, and more specifically in the field of Career Guidance Psychology. This discipline has provided the theoretical and methodological foundation upon which all course content and activities have been conceived and developed (Soresi and Nota, 2020; Savickas et al., 2009). As previously mentioned, the course was launched in the 2018–2019 academic year and on average 110 students per year worked on their Life Design Portfolios. Far from being a merely academic addition, the course represents a psychologically informed educational intervention designed by the authors to address the complex challenges faced by young adults during the uncertain and often destabilizing transition from higher education to professional life. In today’s fluid and competitive labor market, students frequently encounter difficulties in constructing coherent career narratives and maintaining a sense of agency in the face of uncertainty. The Life Design paradigm, which is rooted in social–constructivist and life-span developmental approaches, has been proposed as a preventive and empowering response to these challenges, intended to support the development of adaptability, reflexivity, and self-efficacy through experiential learning and guided reflection. Within this framework, students are encouraged to identify, experiment with, and develop transversal and career-management skills that extend beyond disciplinary boundaries, aligning psychological principles of self-determination and meaning-making with the practical tools of design thinking and strategic foresight. The course thus integrates psychological theory with pedagogical design, supporting opportunities for both personal and professional development. An additional and distinctive aspect of this initiative lies in the natural epistemological affinity between the design profession and the Life Design paradigm. Both share a focus on creativity, project-based reasoning, and the envisioning of possible futures—whether referring to an artifact, a service, or a person’s own life trajectory. Although this course was initially designed for students in Integrated Product Design, many of its core principles and learning activities can be adapted to students from a variety of academic backgrounds. Concepts such as narrative identity, career adaptability, self-reflection, future planning, and the development of transversal skills are not limited to design education. Rather, they provide a theoretically grounded framework that may support students from different fields in reflecting on their personal development and preparing for future professional trajectories. Several activities included in the course, such as the Life Design Portfolio, the My Career Story exercise, reflections on personal values and transferable skills, and the development of a future career project, may be adapted to different educational settings. To make these activities meaningful for students from other disciplines, examples, case studies, and professional scenarios should be contextualized according to their specific areas of study and possible career trajectories. However, some elements of the course are more closely connected to the design field. In particular, the emphasis on visual communication, Portfolio creation, and design thinking approaches reflects characteristics of design education and would require adaptation when introduced in other academic contexts. The implementation of the Life Design approach across different university programs also depends on specific institutional conditions. Collaboration between staff from different disciplines, career guidance professionals, and other university stakeholders may support the integration of reflective activities within curricula. Moreover, recognizing the value of experiential and reflective learning, while providing opportunities for discussion and feedback, may facilitate students’ engagement with the process. Under these conditions, Life Design may represent a flexible framework that can be adapted to different academic contexts while respecting the specific needs, expectations, and professional goals of each discipline. This proximity has facilitated the construction and implementation of the course within a design-oriented academic context and suggests the potential applicability of this psychologically grounded approach beyond design education. Indeed, the Life Design framework is currently being explored as a valuable model in other university contexts, such as guidance activities for academic recognition procedures at the Department of Psychology of the University of Milano-Bicocca, suggesting its adaptability and interdisciplinary potential. Ultimately, this course illustrates one possible way in which principles from the psychology of career guidance and social psychology may be translated into interdisciplinary educational contexts, offering design students—and others—a scientifically grounded framework to cultivate resilience, curiosity, and a sustainable orientation toward their futures. The Life Design course is intended as an educational investment in the holistic development of students as individuals and professionals equipped not only with technical expertise but also with the psychological resources to design and navigate their own evolving life and career paths.

## 6 Limitations and future perspectives

The present paper is limited by its current status as a theory—and practice—driven educational initiative. Although students frequently report positive learning experiences in their Portfolios and narrative reflections, the study lacks a robust empirical framework capable of quantitatively assessing its effectiveness. In particular, no pre–post measures or control groups have been employed to evaluate changes in key psychological resources promoted by the course, such as career adaptability, resilience, future orientation, and self-efficacy. Consequently, there is limited scope to draw causal conclusions regarding the impact of the intervention remains limited. However, this limitation should be interpreted in light of the educational nature of the initiative. The Life Design course is a compulsory curricular component attended by all enrolled students, making the creation of a comparable control group neither practically feasible nor fully consistent with the educational aims of the program. More generally, rigorous experimental designs are often difficult to implement in authentic educational settings where random assignments and strict control of contextual variables are rarely possible (Anderson-Cook, 2005). Furthermore, developmental outcomes are embedded within a broader university experience characterized by multiple educational, relational, and professional influences. As a result, changes in students’ adaptability, resilience, agency, or future perspective cannot be attributed exclusively to participation in the course but rather emerge from the interaction of multiple contextual factors. These methodological challenges are further reinforced by the theoretical assumptions of the Life Design paradigm, which conceptualizes career development as a dynamic process of identity construction, meaning-making, and future projection unfolding over time (Savickas et al., 2009; Guichard, 2016). The developmental trajectories stimulated by the course may continue well beyond its formal duration, making it difficult to identify discrete moments at which change can be conclusively measured. Moreover, educational outcomes related to personal development, agency, and future-oriented thinking are often complex, contextual, and value-laden, and may not be fully captured through standardized indicators alone (Biesta, 2010). Future research should therefore strengthen the empirical foundation of the Life Design course through the adoption of longitudinal, mixed-method, or quasi-experimental designs capable of monitoring students’ developmental trajectories over time while respecting the curricular structure of the intervention. Systematic data collection through surveys, standardized assessments, and longitudinal monitoring would provide a more robust evaluation of changes in students’ transversal competencies and psychological resources. Beyond empirical validation, an important future direction concerns the scalability of the Life Design approach across different educational and disciplinary contexts. An individual’s ability to imagine themselves in the future—a central competence within the Life Design paradigm—has become increasingly relevant in contemporary societies characterized by uncertainty and rapid change. By fostering narrative exploration, meaning-making, adaptability, and self-efficacy in future planning, the Life Design framework holds promising developmental and preventive potential. Its trans-disciplinary nature makes it particularly suitable for application across diverse academic and professional settings, as demonstrated by its emerging use in oriented guidance for academic recognition procedures at the Department of Psychology of the University of Milano-Bicocca. Finally, given the continuously evolving educational and professional landscape, future iterations of the course should remain responsive to contextual changes and theoretical developments within the Life Design paradigm, integrating new psychological perspectives, digital tools, and innovative educational practices. Such efforts may contribute to consolidating the Life Design course as both an empirically grounded and pedagogically relevant resource to support career development and psychological wellbeing in contemporary higher education.

## Data Availability

The original contributions presented in the study are included in the article/supplementary material, further inquiries can be directed to the corresponding author.
